# The long noncoding RNA noncoding RNA activated by DNA damage (NORAD)-microRNA-496-Interleukin-33 axis affects carcinoma-associated fibroblasts-mediated gastric cancer development

**DOI:** 10.1080/21655979.2021.2009412

**Published:** 2021-12-11

**Authors:** Chaoqun Huang, Jiuyang Liu, Liang He, Fubing Wang, Bin Xiong, Yan Li, Xiaojun Yang

**Affiliations:** a Department of Gastrointestinal Surgery, Zhongnan Hospital of Wuhan University, Wuhan, Hubei, China; b Hubei Cancer Clinical Study Center & Hubei Key Laboratory of Tumor Biological Behaviors, Wuhan, Hubei, China; c Department of Breast and Thyroid Surgery, Zhongnan Hospital of Wuhan University, Wuhan, Hubei, China; d Department of Obstetrics and Gynecology, Tongji Hospital, Tongji Medical College, Huazhong University of Science and Technology, Wuhan, Hubei, China; e Department of Peritoneal Cancer Surgery, Beijing Shijitan Hospital, Capital Medical University, Beijing, China

**Keywords:** Lncrna NORAD, miR-496, IL-33, gastric cancer, carcinoma-associated fibroblasts

## Abstract

Carcinoma-associated fibroblasts (CAFs) are one of the crucial parts of in the tumor microenvironment and contribute to tumor progression. Interleukin-33 (IL-33), a tissue-derived nuclear cytokine from the IL-1 family, has been found abnormally expressed in tumor cells and Fibroblast. However, the role and mechanism of IL-33 in the interaction between gastric cancer (GC) cells and CAFs need investigation. Presently, we inquire into the function of lncRNA NORAD-miR-496 axis-mediated IL-33 in modulating the GC-CAFs interaction. Real-time reverse transcription-polymerase chain reaction (RT-PCR) was adopted to gauge the expression of NORAD, miR-496, and IL-33 in GC tissues and cells, and gain- or loss-of-function assays were conducted to investigate the role of them in GC. A GC cell-CAFs co-culture model was established to explore the interaction between CAFs and GCs. As exhibited, NORAD was up-regulated in GC tissues and cells, while miR-496 was remarkably down-regulated. Overexpressing NORAD substantially promoted the proliferation, migration, invasion, and EMT of GC cells and repressed cell death, while overexpressing miR-496 had the opposite effects. Additionally, NORAD enhanced the IL-33 expression and the release of IL-33 from GC cells. The dual-luciferase reporter assay confirmed that miR-496 was a target of NORAD and targeted IL-33. CAFs aggravated the malignant behaviors of GC cells as indicated by both experiments. However, NORAD knockdown in CAFs reversed CAFs-mediated promotive effects on GC cells. In conclusion, NORAD enhanced the promotive effect of CAFs in GC cells by up-regulating IL-33 and targeting miR-496, which provided new insights into the microenvironment of GC cells and CAFs.

**Abbreviation** ANOVA: Analysis of Variance; BCA:Bicinchoninic acid; CAFs: carcinoma-associated fibroblasts; CCK-8: cell counting kit-8; ceRNA: competing endogenous RNA; DAPI: 4′,6-diamidino-2-phenylindole; DMEM: Dulbecco’s minimal essential medium/Ham’s; ECL: enhanced chemiluminiscent; ELISA: Enzyme-Linked Immunosorbent Assay; EMT: epithelial-mesenchymal transition; FBS: fetal bovine serum; FISH:Fluorescence in situ hybridization; FITC:fluorescein isothiocyanate; FSP:fibroblast-specific protein; GAPDH: glyceraldehyde-3-phosphate dehydrogenase; GC: gastric cancer; IHC: immunohistochemistry; IL: Interleukin; lncRNA: long Noncoding RNA; miR-496: microRNA-496; MMP-14:matrix metalloproteinase-14; MUT:mutant; MYH9: myosin heavy chain 9; NFs: normal fibroblasts; NORAD: Noncoding RNA activated by DNA damage; ORF: open reading frame; PBS: phosphate-buffered saline; PMSF: Phenylmethylsulfonyl fluoride; PVDF: polyvinylidene difluoride; RIPA: Radio-Immunoprecipitation Assay; RT-PCR: Real-time reverse transcription polymerase chain reaction; S100A4:S100 calcium binding protein A4; SDS-PAGE: sodium dodecyl sulfate-polyacrylamide gel electrophoresis; sh-NC: short-hairpin RNA negative control; sh-NORAD: short-hairpin RNA of NORAD; α-SMA: α-smooth muscle actin; TBST: Tris-buffered saline with Tween-20; TGF-β1: Transforming growth factor β1; TUNEL: TdT-mediated dUTP Nick-End Labeling; TWIST1: the twist-related protein 1; VEGF-C: vascular endothelial growth factor C; WT: Wildtype.

## 1. Introduction

Gastric cancer (GC) has become a global concern, and it is one of the leading causes of death in China [1]. Recent advances in the main treatments for GC, such as surgery, chemotherapy and radiotherapy, have significantly prolonged the survival rate of GC patients. In addition to those traditional treatments, cancer immunotherapy has has become a powerful clinical strategy for treating cancer [2,3]. Nevertheless, the metastasis and recurrence rate within five years after radical resection remains high, with the median time to short-term recurrence ranging from 14 months to 29 months [4]. Therefore, it is crucial to study molecular markers for the early diagnosis of GC and develop new therapeutic methods.

Peritoneal metastasis is a vital step in the progression of GC [5]. In this process, carcinoma-associated fibroblasts (CAFs), which are originated from the intrinsic fibroblasts, become one of the most crucial components of the tumor microenvironment [6]. CAFs can interact with tumor cells through various cytokines, thus affecting tumorigenesis, tumor cell proliferation, invasion, metastasis, angiogenesis, epithelial-mesenchymal transition (EMT), tumor stemness maintenance, and drug resistance [7,8]. Hence, exploring the role of CAFs in modulating GC progression is helpful in elucidating GC development.

Long noncoding RNAs (lncRNAs) are more than 200 nucleotides long. LncRNAs cannot be translated into proteins due to the lack of an independent open reading frame (ORF). Nevertheless, they can regulate gene expression in diversified ways, such as epigenetic modifications, nuclear translocation, gene transcription and mRNA translation [9]. Increasing studies have confirmed that lncRNAs act as oncogenes or tumor suppressor genes to modulate the occurrence and development of tumors. These abnormally expressed lncRNAs can not only be used as diagnostic markers of malignancies but also are potential therapeutic targets [10]. Additionally, multiple lncRNAs are found involved in the proliferation and metastasis GC [11]. Of special note, noncoding RNA activated by DNA damage (NORAD) is a novel lncRNA derived from segment q11.23 of chromosome 20. Interestingly, NORAD has been verified as an oncogene in several tumors, including pancreatic cancer [12], colorectal cancer [13] and GC [14,15]. Thus, we suppose that NROAD affects the interaction between GC and CAFs.

miRNAs are a class of single-stranded noncoding RNA with a length of about 22–25 nucleotides. Circulating miRNAs have been identified as biomarkers for tumor screening due to their high stability and rich content [16]. Like lncRNAs, miRNAs can also regulate the expression of tumor-related genes at the post-transcriptional or translational level [17,18]. Additionally, various miRNAs are abnormally expressed during GC development. For example, miR-425 and miR-532, which are up-regulated in GC, boost GC cells’ proliferation, migration, and invasion [19,20]. In contrast, miR-320a restrains the development of GC [21].

Recently, accumulating studies have suggested that lncRNAs function as a competing endogenous RNA (ceRNA) via targeting miRNAs, forming a lncRNA-miRNA-mRNA regulatory axis [22]. Here, we discovered that NORAD was up-regulated in GC tissues, which enhanced GC cells’ proliferation and metastasis by regulating the miR-496/IL-33 axis. Meanwhile, CAFs promoted the malignant behaviors of GC cells. Supported by those results, we guessed that the NROAD-miR-496-IL-33 axis plays a role in GC-CAFs microenvironment. Thus, we further explored the functions of NORAD-mediated CAFs on GC progression. In conclusion, this study explored the role and mechanism of NORAD in regulating GC cell proliferation and metastasis and CAF-GC interaction, bringing new hope for targeted therapy of GC.

## 2. Materials and methods

### 2.1 Specimens

Forty-four pairs of GC tissues and matched non-tumor specimens were harvested from patients who underwent surgery in Zhongnan Hospital of Wuhan University from June 2017 to June 2018. All tissues were pathologically confirmed by two pathologists. None of the patients received chemo/radiotherapy or biotherapy prior to surgery. All samples were frozen in liquid nitrogen within 5 mins after resection and then sorted at −80°C until further use for the NORAD level analysis. The cutoff value 1 was set as distinguishing low or high NROAD expression in GC tissues. This study was granted by the Research Ethics Committee of the Zhongnan Hospital of Wuhan University, with informed consent was signed by all patients.

### 2.2 Isolation and culture of primary CAFs

Normal gastric tissues or GC tissues were taken and placed in a 4°C pre-cooled PBS. The tissues were cut into sections and trypsinized by collagenase IV (Invitrogen, CA, United States) at 37°C for 3 hours. Then the trypsinized sections were filtrated through a 70-μm mesh. After that, the filtrate was transferred into an EP tube, centrifugated (200 rpm for 10 min) and rewashed by PBS 3 times to remove red blood cells. Next, the collected cells were grown in the DMEM culture medium comprising 10% FBS (FBS, HyClone, Logan, UT, USA) and cultured in an incubator (37°C, 5% CO_2_, 95% humidity). After 3 hours, the adherent cells were pooled and cultured in other dishes to obtain CAFs and NFs [23].

### 2.3 Cells and cell culture

Human GC cell lines (AGS, MGC-803, BGC-823, and SGC-7901) and normal gastric epithelial cells GSE-1 were ordered from American Type Culture Collection (ATCC, Rockville, MD, USA). The culture medium was the DMEM-F12 medium in which 10% fetal bovine serum (FBS, HyClone, Logan, UT, USA) and 1% penicillin/streptomycin were present. All cells were cultured with 100% humidity and 5% CO_2_ at 37°C, with the culture medium altered every 2 to 3 days.

### 2.4 Cell co-culture model

NFs, CAFs and GC cells (AGS and SGC-7901) at the logarithmic growth stage were taken, and a cell co-culture model was established in 24-well plates by using transwell chambers (Corning, 0.4 μm in diameter) [24]. Briefly, NFs or CAFs (1 × 10^5^) were inoculated in the upper chamber, and GC cells were seeded in 24-well plates (below the chambers) at 5 × 10^5^/well. The medium was DMEM-F12 medium with 10% FBS (HyClone, Logan, UT, USA) and 1% penicillin/ streptomycin. After 24 hours of co-culture, GC cells were employed for further experiments. Also, the co-culture medium was collected and centrifugated for removing cells, and the level of IL-33 was determined.

### 2.5 Cell viability detection

GC cells (AGS and SGC-7901) co-cultured with or without NFs/CAFs were taken and resuspended by a complete culture medium. Then 100 μL cell suspension was added to the 96-well plates (about 4 × 10^3^ cells/well). After different periods (24, 48, 72, and 96 hours) of culturing in an incubator at 37°C with 5% CO_2_ and 100% humidity, 10 μL CCK-8 solution (Beyotime, Shanghai, China) was spiked into each well, and the plates were incubated at 37°C for 4 hours. After that, the absorbance of each well was gauged at 450 nm with a Thermomax microplate reader [25].

### 2.6 Cell transfection

GC cells (AGS and SGC-7901) were inoculated in 12-well plates (1 × 10^6^ cells per well). NORAD overexpression plasmids, short-hairpin RNA of NORAD (sh-NORAD), small interfere RNA of IL-33 (si-IL-33), miR-496 mimics and the negative controls (GenePharma, Shanghai, China) were respectively transfected into the cells by utilizing the Lipofectamine 2000 reagent (Thermo Fisher Science, Waltham, MA, USA) as per the manufacturer’s directions. Forty-eight hours after the transfection, the culture medium was exchanged with a fresh one. Real-time reverse transcription-polymerase chain reaction (RT-PCR) was performed to verify the NORAD and miR-496 expression for confirming the transfection efficiency.

### 2.7 Real-time reverse transcription-polymerase chain reaction (RT-PCR)

GC cells or CAFs were harvested, and the total RNA was extracted out of cells using the TRIzol reagent (Invitrogen). Thermo Nano Drop 2000 was adopted to detect the concentration and purity of total RNA, and SDS-PAGE Agilent-2100 was applied to examine the integrity of total RNA. The total RNA was reversely transcribed into cDNAs with the aid of the RevertAid First Strand cDNA synthesis kit (Fermentas, Waltham, MA, USA). Roche FastStart Universal SYBR Green Master was utilized for RT-PCR, which was performed with 45 cycles of initial denaturation (95°C, 30s), denaturation (95°C, 5s), and primer annealing (60°C, 30s). The 2^−ΔΔCT^ method was adopted to check the relative expression of various RNAs [26]. GAPDH acted as a housekeeping gene of the other genes. Primer5.0 was applied to design primers, and the primer sequences are as follows: *NORAD* Forward: 5ʹ-CAGAGGAGGTATGCAGGGAG-3ʹ, Reverse: 5ʹ- GGATGTCTAGCTCCAAGGGG −3ʹ; *miR-496*: Forward 5ʹ-GCTGAGTATTACATGGCCAATCTC-3ʹ, *IL-33* Forward: 5ʹ-TCCCAACAGAAGGCCAAAGA-3ʹ, Reverse: 5ʹ-AAAGGCAAAGCACTCCACAG-3ʹ; *TGFβ1*, Forward: 5ʹ- TACAGCAACAATTCCTGGCG-3ʹ, Reverse: 5ʹ- CGTTGATGTCCACTTGCAGT-3ʹ; *TWIST1*, Forward: 5ʹ-AGTCTTACGAGGAGCTGCAG-3ʹ, Reverse: 5ʹ- AGGAAGTCGATGTACCTGGC-3ʹ; *MMP-14*, Forward: 5ʹ- TGTCTTCAAGGAGCGATGGT-3ʹ, Reverse: 5ʹ-GCTCCTTAATGTGCTTGGGG-3ʹ; *VEGF-C*, Forward: 5ʹ-TAGACGTTCTCTGCCAGCAA-3ʹ, Reverse: 5ʹ-GGTGTCTTCATCCAGCTCCT-3ʹ; *GAPDH*: Forward: 5ʹ- CTGACTTCAACAGCGACACC-3ʹ, Reverse: 5ʹ- GTGGTCCAGGGGTCTTACTC-3ʹ.

### 2.8 Western blot (WB)

NFs, CAFs and GC cells (AGS and SGC-7901) were harvested and lysed by the RIPA (containing 1% PMSF) lysis solution (Beyotime, Shanghai, China), followed by the total protein extraction with centrifugation (14000 rpm for 30 min). The BCA protein concentration kit (Beyotime, Shanghai, China) was applied for protein concentration quantification. Then, the proteins were isolated via SDS-PAGE and transferred to PVDF membranes at a constant current of 200 mA for 90 min. After being sealed with 5% skim milk solution for 2 hours, the PVDF membranes were incubated with primary antibodies of anti-E-cadherin (Abcam, ab1416, 1:1000) and anti-Vimentin (Abcam, ab8978, 1:1000) overnight at 4°C. Subsequently, the membranes were flushed with TBST three times (15 min each time) and maintained with the secondary antibodies at room temperature (RT) for 2 hours. After cleaning with TBST again, the protein bands were exposed using an ECL solution (Beijing Xinjingke Biotechnologies Co., Ltd, China). The densitometry analysis was performed using Image J 3.0 (IBM, USA), and GAPDH served as a control.

### 2.9 Enzyme-Linked Immunosorbent Assay (ELISA)

The co-culture medium of CAFs-GC cells was collected and centrifuged at 4°C at 1000 rpm for 10 min to remove cells and debris. The IL-33 level in the supernatant was assessed with the Human IL33 ELISA Kit (Abcam, ab223865) in strict accordance with the instructions provided by the manufacturer [27]. The experiment was repeated three times, with three repetitive wells in each experiment.

### 2.10 Transwell assay

The transwell assay was conducted to examine cell migration and invasion [28]. After transfection for 24 hours, GC cells were collected and resuspended by serum-free DMEM-F12 medium at a cell density of 1 × 10^5^ cells/ mL. The cells were inoculated in the upper chamber of the transwell with 8 μm pores. In contrast, the lower chamber of the transwell contained DMEM-F12 medium with 500 μL 10% PBS, in which the cells were cultured for 6 hours. Unmigrated cells on the upper membrane were wiped off, and cells that migrated and adhered to the lower chamber were secured with 4% paraformaldehyde and stained with crystal violet. The number of cells in five random fields of view were counted using a light microscope, and the mean value of three repetitive wells was taken to represent the migration of tumor cells. For the invasion assay, the chambers were pre-coated with Matrigel (USA, BD), and the other steps were the same as the migration assay.

### 2.11 Fluorescence in situ hybridization (FISH)

The FISH assay was performed following the instructions of the FISH kit (RiboBio, Guangzhou, China) to testify lncRNA NORAD expression in GC cells [29]. The NORAD FISH probes were designed and synthesized by RiboBio (Guangzhou, China). Briefly, 4% formalin was applied to fix GC cell line SGC-7901 and normal gastric epithelial cells GSE-1 for 15 min. Afterward, the cells got prehybridized in PBS and hybridized at 37°C for 30 min in a hybridization solution. DAPI (4′,6-diamidino-2-phenylindole) staining (Beyotime, China) was employed to label the cell nuclei. Both GC cell and gastric epithelial cells were observed and photographed with a microscope (Carl Zeiss, Oberkochen, Germany).

### 2.12 TdT-mediated dUTP Nick-End Labeling (TUNEL) assay

The In Situ Cell Death Detection Kit (cat. no. 11684817910, Roche, Basel, Switzerland) was applied for the determination of unspecified cell death. All procedures were performed following the manufacturer’s directions. GC cells were immobilized by 4% paraformaldehyde for 15 min, rinsed in PBS for 15 min, followed by double-staining with TUNEL staining reagent and DAPI dyeing (Beyotime, Shanghai, China) [30]. A fluorescent microscope (Olympus Corporation; magnification, ×400) was employed to observe the fluorescence images. Experiments were performed in triplicate.

### 2.13 Immunofluorescence staining

To detect IL-33 in GC cells and S100A4 in NFs/CAFs, cellular immunofluorescence staining was performed. Briefly, the cells seeded in the culture plates were fastened by 4% paraformaldehyde for 15 min, rinsed in PBS for 15 min, permeabilized with 0.1% Triton X-100 for 10 min, and blocked with 5% goat serum for 1 hour at RT. Next, they were incubated with the primary antibodies, including anti-IL-33 (ab187060, Abcam, 1:200) and anti-S100A4 (ab124805, Abcam, 1:200) for 12 hours at 4°C. Afterward, they were subjected to PBS washing 3 times (5 min each time) and incubated with a Cy3- or FITC-conjugated goat anti-rabbit IgG (Beyotime, Shanghai, China) at RT for 1 hour. The nucleus was stained with DAPI (Beyotime, Shanghai, China) [31]. A fluorescent microscope (Olympus Corporation; magnification, ×400) was adopted to observe the fluorescence images. Experiments were performed in triplicate.

### 2.14 In-vivo *experiment*

AGS cells were co-cultured with CAF transfected with or without si-NORAD in a transwell co-culture model for 48 hours. Then, AGS cells were harvested and adjusted to reach a density of 1 × 10^7^cells/mL. Next, 100 μL of single-cell suspension was slowly injected subcutaneously into the sterilized right forelimb of the mice using a 1 mL syringe. The tumor length and diameter were measured *in vivo* with calipers for each group of rats and then injected intratumorally once every seven days. The volume of the tumor is equal to length×width^2^/2 [32]. After 4 weeks, mice were sacrificed with an overdose of phenobarbital (100 mg/kg), and tumor tissues were carefully peeled, weighed and photographed. Subsequently, the expression of KI67, E-cadherin, Vimentin, IL-33 in tumor tissues was gauged by immunohistochemistry (IHC). The study was granted by the Medical Ethics Committee of the Zhongnan Hospital of Wuhan University.

### 2.15 Immunohistochemistry

Mouse tumor tissues were harvested, secured in 4% paraformaldehyde at RT for 24 hours, routinely dehydrated, visualized, waxed, embedded, and sectioned (5 μm). Then microwave repair was implemented for 1 min, followed by 3% H_2_O_2_ inactivation for 15 min and 5% BSA closure for 30 min. After shaking off the excess liquid, rabbit anti-Rat KI67, E-cadherin, Vimentin, and IL-33 polyclonal antibodies (all calibrated 1:100) were added dropwise and maintained overnight at 4°C. The following day, a drop of horseradish peroxidase-tagged goat anti-rabbit IgG was added and incubated for 30 min, followed by the incubation of DAB solution for 40 min and DAB color rendering for 5 min. After termination of the reaction, the sections were dehydrated, transparentized, sealed with neutral gum and placed under the microscope for observation [31].

### 2.16 Data analysis

All data in this study were processed by SPSS20.0 statistical analysis software (SPSS Inc., Chicago, IL, USA). Measurement data were represented as ‘mean ± standard deviation’ (x± sd). Statistical differences between groups were analyzed using one-way and two-way ANOVAs followed by Tukey’s multiple comparisons test. *P* value< 0.05 was considered statistically significant.

## 3 Results

### 3.1 NORAD was up-regulated in GC, which aggravated the malignant behaviors of GC cells

**Figure 1.**
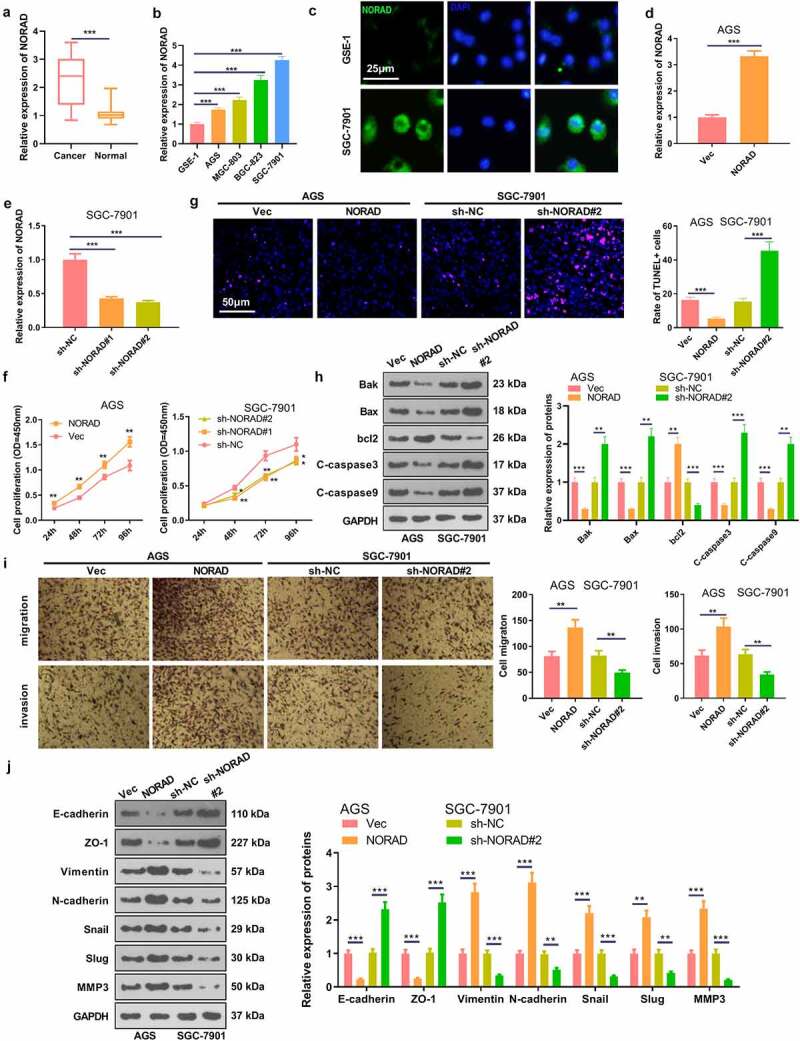
NORAD was up-regulated in GC tissues and cells, which accelerated GC progression a. RT-PCR chacked NORAD expression in GC tissues and adjacent normal tissues. *** represents *P* < 0.001. b. RT-PCR examined the NORAD profile in normal gastric epithelial cells GSE-1 and GC cell lines AGS, MGC-803, BGC-823 and SGC-7901; N = 3, *** represents *P* < 0.001 (vs. GSE-1 group). c. FISH was performed to determine the expression and location of NORAD in GSE-1 and SGC-7901 cells. d and e. AGS cells were transfected with NORAD overexpression plasmids and SGC-7901 cells were transfected with sh-NORAD or sh-NC. The NORAD level in the cells was tested by RT-PCR. f. CCK-8 was adopted to verify AGS and SGC-7901 cell viability. g. TUNEL was carried out to evaluate cell death. h. Expression of Bak, Bax, bcl2, Caspase3 and Caspase9 in AGS and SGC-7901 cells was monitored by WB. i. Transwell assay measured cell migration and invasion. j. WB checked the expression of EMT-related markers E-cadherin, ZO-1, Vimentin, N-cadherin, Snail, Slug and MMP3 in GC cells respectively. * represents *P* < 0.05, ** represents *P* < 0.01, *** represents *P* < 0.001 (vs. Vec or sh-NC group). N = 3

For investigating the role of NORAD in GC, the NORAD expression in GC tissues and cells was assessed. The results revealed that the NORAD profile was notably enhanced in GC tissues versus adjacent non-cancerous tissues Figure 1(a). Higher NORAD level was significantly associated with tumor sizes and clinical stages (Table 1). Also, a remarkable up-regulation of NORAD in GC cell lines (including AGS, MGC-803, BGC-823 and SGC-7901) was observed versus normal gastric epithelial cells, Figure 1(b). Moreover, we performed the FISH assay to determine the expression and location of NORAD in the cells. It was discovered that NORAD was mainly distributed in the cytoplasm of GC cell line SGC-7901, and it was present at higher levels in GC cells versus normal gastric epithelial cells GES-1 Figure 1(c). Next, NORAD overexpression and knockdown models were set up to figure out the impact of NORAD on GC cells Figure 1(d,e). As hinted by CCK-8 data, overexpressing NORAD prominently enhanced AGS cell proliferation, while knocking down NORAD notably curbed SGC-7901 cell proliferation Figure 1(f). The dyed GC cells were determined by the TUNEL staining, which displayed that NORAD overexpression reduced cell apoptosis, while down-regulating NORAD enhanced cell apoptosis Figure 1(g). WB outcomes displayed that overexpressing NORAD elevated the expression of the anti-apoptotic protein bcl2 and lowered the expression of the pro-apoptotic proteins Bak, Bax, c-Caspase-3 and c-Caspase-9 in AGS cells. In contrast, knocking down NORAD restrained the expression of bcl2 and boosted the expression of Bak, Bax, Caspase-3 and Caspase-9 in SGC-7901 cells Figure 1(h). Further detection of cell migration and invasion substantiated that overexpressing NORAD heightened GC cell migration and invasion, while NORAD knockdown exerted the opposite effect Figure 1(i). In addition, WB was conducted to measure the expression of EMT-related markers E-cadherin, ZO-1, Vimentin, N-cadherin, Snail, Slug and MMP3 in GC cells. As a result, overexpressing NORAD facilitated the expression of Vimentin, N-cadherin, Snail, Slug and MMP3 and prominently retarded the expression of E-cadherin and ZO-1. In parallel, knocking down NORAD had the opposite effect Figure 1(j). These results confirmed that NORAD accelerated GC development.

**Table 1.** Correlation between NORAD expression and clinicopathological features in patients with GC

		NORAD expression		
Features	Cases	Low (n = 22)	High (n = 22)	*P* value
Gender				
Male	30	16	13	0.34
Female	14	6	9	
Age				
	20	12	11	0.763
≥60	24	10	11	
Tumor size				
<5 cm	27	17	10	0.0302
≥5 cm	17	5	12	
T stage				
T1-T2	26	17	9	0.0142
T3-T4	18	5	13	
N stage				
N0-N1	27	14	13	0.461
N2-N3	15	6	9	
Distant metastases				
Yes	19	7	12	0.128
No	25	15	10	

* represents p < 0.05.

### 3.2 NORAD up-regulated IL-33 and IL-33 knockdown induced inhibitive effects in GC

**Figure 2.**
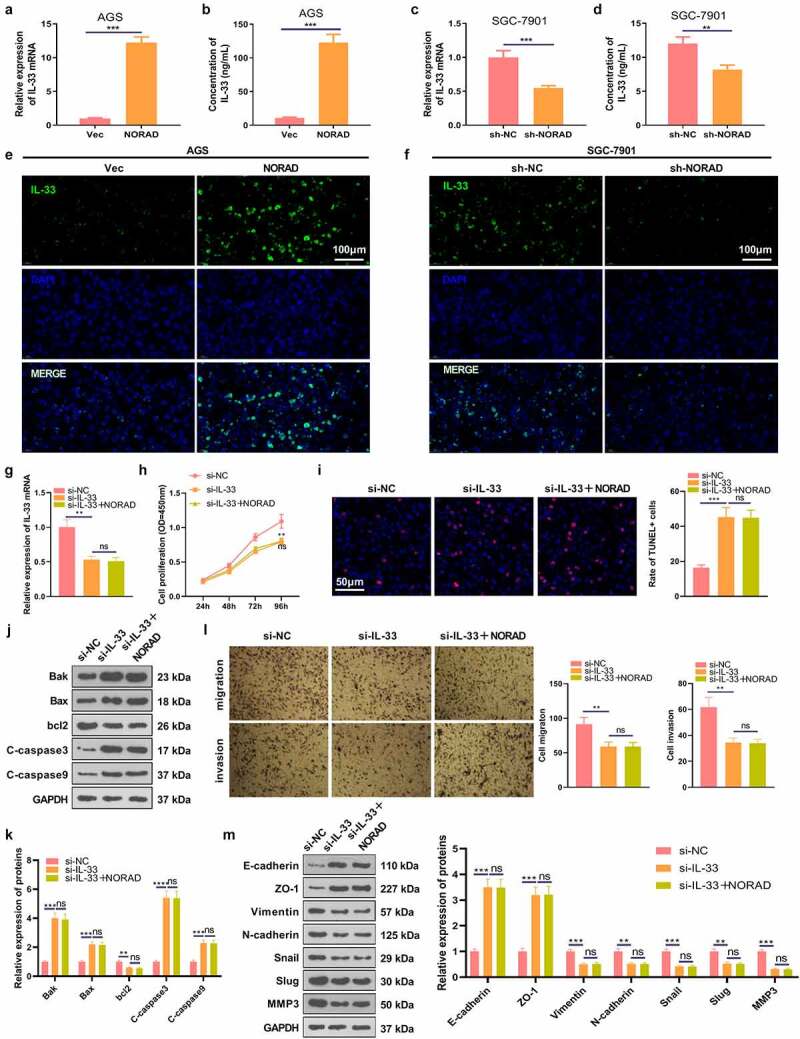
NORAD promoted the expression of IL-33. a and c. RT-PCR detected the mRNA expression of IL-33 in GC cells (AGS and SGC-7901). b and d. ELISA validated IL-33 expression in the cell culture medium. E-F. Cellular immunofluorescence was used to detect IL-33 (marked by green color) in AGS and SGC-7901 cells. g. RT-PCR tested the level of IL-33 mRNA in AGS cells. H. AGS cell proliferation was assayed by CCK-8. I. TUNEL assessed AGS cell apoptosis. j and k. WB was conducted for the detection of Bak, Bax, bcl2, Caspase3 and Caspase9 expression in AGS cells. L.Transwell assay verified AGS cell migration and invasion. M. WB was implemented to check the expression of EMT-related markers (E-cadherin, ZO-1, Vimentin, N-cadherin, Snail, Slug and MMP3) in AGS cells. ns represents *P* > 0.05, *represents *P* < 0.05, ** represents *P* < 0.01, *** represents *P* < 0.001, ****represents *P* < 0.0001. N = 3

Previous studies have established that IL-33, a vital cytokine, plays a significant role in regulating malignant phenotypes of various tumors [33,34]. In this study, we also wondered whether IL-33 was affected by NORAD in GC. Our findings testified that overexpressing NORAD observably elevated the mRNA and protein expression of IL-33, while knocking down NORAD substantially curbed IL-33 expression Figure 2(a,d). In addition, the cellular immunofluorescence result indicated that NORAD enhanced IL-33 expression Figure 2(e,f). The si-NC, si-IL-33, si-IL-33 and NORAD overexpression plasmids were transfected into AGS cells for 24 hours and RT-PCR was performed. It turned out that knockdown of IL-33 choked IL-33 expression in AGS cells versus the si-NC group, while there was no major disparity in IL-33 expression in AGS cells between the si-IL-33 group and the si-IL-33 + NORAD group Figure 2(g). As hinted by CCK-8 data, knocking down IL-33 restrained AGS cell proliferation versus the si-NC group. In parallel, the si-IL-33 and si-IL-33+ NORAD groups had almost identical levels of AGS cell proliferation Figure 2(h). TUNEL and WB results displayed that knocking down IL-33 boosted apoptosis, up-regulated pro-apoptotic proteins and down-regulated anti-apoptotic proteins in AGS cells versus the si-NC group. Nonetheless, there was no significant difference in AGS cell apoptosis between the si-IL-33 group and the si-IL-33 + NORAD group Figure 2(i-k). Additionally, the Transwell assay exhibited that knocking down IL-33 attenuated the migrative and invasive ability of AGS cells versus the si-NC group. Nevertheless, the migration and invasion of AGS cells in the si-IL-33 + NORAD group were not significantly altered versus the si-IL-33 group Figure 2(l). Also, WB outcomes uncovered that knocking down IL-33 fostered the expression of E-cadherin and ZO-1 and repressed the expression of Vimentin, N-cadherin, Snail, Slug and MMP3 versus the si-NC group. In contrast, there was no significant difference in the expression of these EMT-related protein markers in AGS cells in the si-IL-33 group versus the si-IL-33 + NORAD group Figure 2(m). Thus, IL-33 was positively regulated by NORAD, and knocking down IL-33 curbed GC progression *in vitro*.

### 3.3 miR-496 was a target for NORAD and targeted IL-33

**Figure 3.**
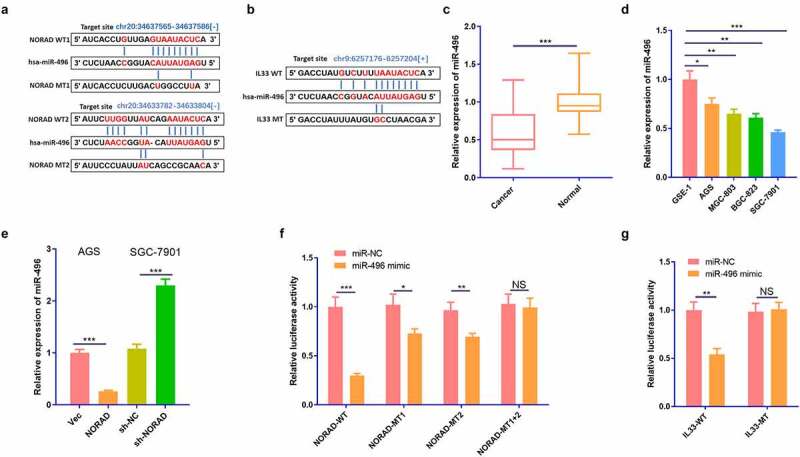
miR-496 was the target of NORAD and targeted IL-33. a and b. The Starbase (http://starbase.sysu.edu.cn/index.php) was employed to predict the targets of NORAD and IL-33. The binding sites between NORAD and miR-496, miR-496 and IL-33 were shown. c. RT-PCR checked the expression of miR-496 in GC tissues and the adjacent normal tissues. d. RT-PCR examined the expressions of NORAD in normal gastric epithelial cells GSE-1 and GC cell lines AGS, MGC-803, BGC-823 and SGC-7901. e. RT-PCR tested the expression of miR-496 in AGS and SGC-7901 cells. F and g. Dual-luciferase reporter assay was used to verify the relationship between NORAD and miR-496, as well as between miR-496 and IL-33. NS represents *P* > 0.05, * represents *P* < 0.05, ** represents *P* < 0.01, *** represents *P* < 0.001. N = 3

Inspired by the lncRNA-miRNA-mRNA regulatory network, we predicted the targets of NORAD and IL-33 through the Starbase database (http://starbase.sysu.edu.cn/index.php.). The results revealed that miR-496 was not only a ceRNA of NORAD but also potentially targeted IL-33 Figure 3(a,b). We adopted RT-PCR to monitor the expression of miR-496 in GC tissues and cells, discovering that miR-496 was down-regulated in both GC tissues (compared with normal adjacent tissues) and GC cells (compared with GSE-1 cells) Figure 3(c,d). In addition, overexpressing NORAD signally hindered the miR-496 profile, while knocking down NORAD elevated the miR-496 expression Figure 3(e). As corroborated by the dual-luciferase reporter assay, miR-496 declined the luciferase activity of AGS cells transfected with NORAD-WT vectors and IL-33-WT vectors. However, it had little influence on the luciferase activity of AGS cells transfected with NORAD-MUT vectors or IL-33-MUT vectors Figure 3(f,g). Thus, miR-496 was targeted by NORAD and targeted IL-33.

### 3.4 Overexpressing miR-496 restrained GC cells’ proliferation and invasion and lowered the IL-33 expression

**Figure 4.**
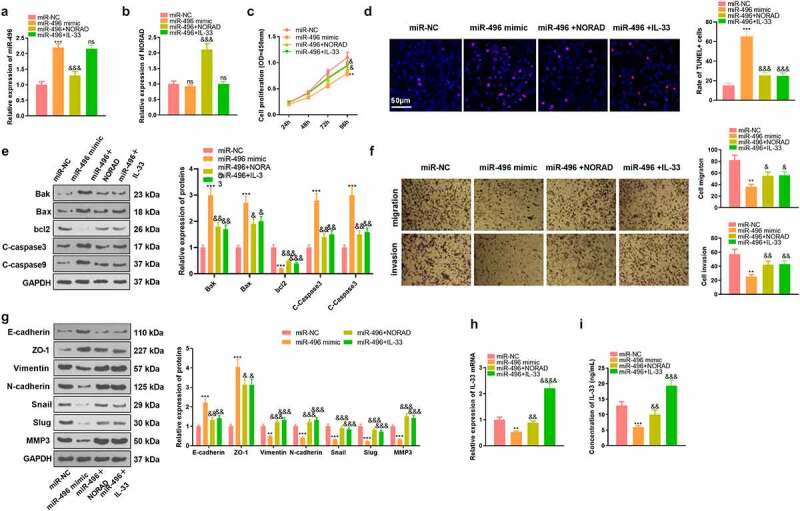
Overexpressing miR-496 curbed GC cells’ proliferation and invasion and declined the IL-33 expression. AGS cells were transfected with miR-496 mimics and/or NORAD, IL-33 overexpression plasmids. a-b. Expression of miR-496 and NORAD in AGS cells was detected by RT-PCR. c. CCK-8 tested the proliferation of AGS cells. d. TUNEL assay gauged unspecified cell death. e. Expression of Bak, Bax, bcl2, Caspase3 and Caspase9 in AGS cells was gauged by WB. f. Transwell assay tested the migrative and invasive abilities of AGS cells. g. WB examined the expression of EMT-related markers E-cadherin, ZO-1, Vimentin, N-cadherin, Snail, Slug, and MMP3 in GC cells respectively. h and i. RT-PCR and ELISA were adopted to monitor the expression changes of IL-33 mRNA and protein. ns, *,**, *** represents *P* > 0.05, *P* < 0.05, *P* < 0.01, *P* < 0.001 vs. miR-NC group, respectively; ns,&, &&, &&&, &&&& represents *P* > 0.05, *P* < 0.05, *P* < 0.01, *P* < 0.001, *P* < 0.0001 vs. miR-496 mimic group, respectively; N = 3

To further explore the role of miR-496 and IL-33 in regulating the biological behaviors of GC cells, we transfected AGS cells with miR-NC, miR-496 mimics, miR-496 mimics+NORAD overexpression plasmids, and miR-496 mimics+IL-33 overexpression plasmids. Notably, up-regulated miR-496 was repressed by NORAD. Meanwhile, miR-496 had no substantial influence on the NORAD expression, and IL-33 had little impact on miR-496 and NORAD expression in GC cells Figure 4(a,b). Cell proliferation, apoptosis, migration and invasion were gauged using CCK-8, TUNEL, WB and Transwell. As a result, overexpressing miR-496 notably depressed the proliferation, migration and invasion of AGS cells and contributed to apoptosis Figure 4(c-f). Also, WB outcomes disclosed that overexpression of miR-496 facilitated the expression of E-cadherin and ZO-1 and declined the expression of Vimentin, N-cadherin, Snail, Slug and MMP3 Figure 4(g). RT-PCR results uncovered that overexpressing miR-496 markedly down-regulated IL-33 Figure 4(h,i). What is more, up-regulation of NORAD or IL-33 on the basis of miR-496 overexpression resulted in a significant enhancement of proliferation, migration and invasion of AGS cells and a pronounced reduction in apoptosis, along with the up-regulation of IL-33 Figure 4(c-i). These results hinted that miR-496 delayed GC evolvement, whereas this effect could be attenuated by NORAD or IL-33.

### 3.5 CAFs intensified the malignant behaviors of GC cells

**Figure 5.**
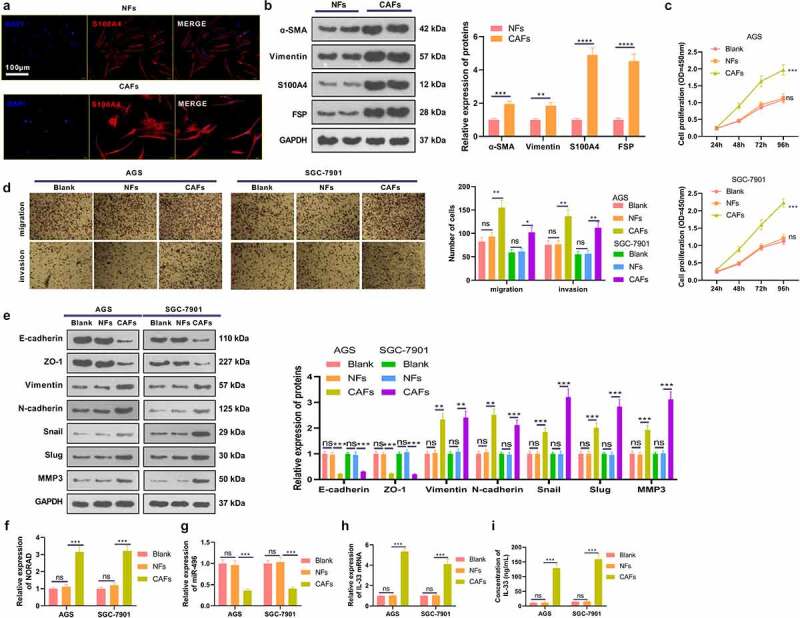
CAFs intensified the malignant behaviors of GC cells a. Both NFs and CAFs were isolated, and cellular immunofluorescence was carried out to identify the NFs and CAFs (marked by S110A4). b. WB verified fibroblast markers α-SMA, Vimentin, FSP and S110A4 in NFs and CAFs. NFs and CAFs were co-cultured with GC cells (AGS and SGC-7901). c. CCK-8 validated the proliferation of AGS and SGC-7901 cells. d. Transwell assay checked the migrative and invasive abilities of AGS and SGC-7901 cells. e. WB monitored the expression of EMT-related markers E-cadherin, ZO-1, Vimentin, N-cadherin, Snail, Slug and MMP3 in GC cells. f-h. RT-PCR gauged the NORAD/miR-496/IL-33 expression in AGS and SGC-7901 cells. i. ELISA detected IL-33 profiles in the culture medium. N = 3. ns, *,**, *** represents *P* > 0.05, *P* < 0.05, *P* < 0.01, *P* < 0.001

In the tumor microenvironment, CAFs can further stimulate the proliferation, metastasis and angiogenesis of GC cells [35]. We isolated both NFs and CAFs, which were identified by S110A4-cellular immunofluorescence Figure 5(a). WB data substantiated that CAFs had enhanced expression of α-SMA, Vimentin, FSP and S110A4, all of which were fibroblast markers Figure 5(b). We then co-cultured NFs and CAFs with two GC cells (AGS and SGC-7901) and checked the malignant behaviors of GC cells. As a result, NFs had no substantial influence on the proliferation, migration, invasion and EMT of GC cells, while CAFs prominently enhanced those behaviors of GC cells Figure 5(c-e). Furthermore, the NORAD-miR-496-IL-33 axis in GC cells was examined. We observed that co-culture of GC cells with CAFs resulted in significantly enhanced levels of NORAD and IL-33, with miR-496 being suppressed Figure 5(f-i). Collectively, CAFs accelerated GC progression via affecting the NORAD-miR-496-IL-33 axis in GC cells.

### 3.6 Knocking down NORAD abated CAFs-mediated promotive effects on GC cell proliferation

**Figure 6.**
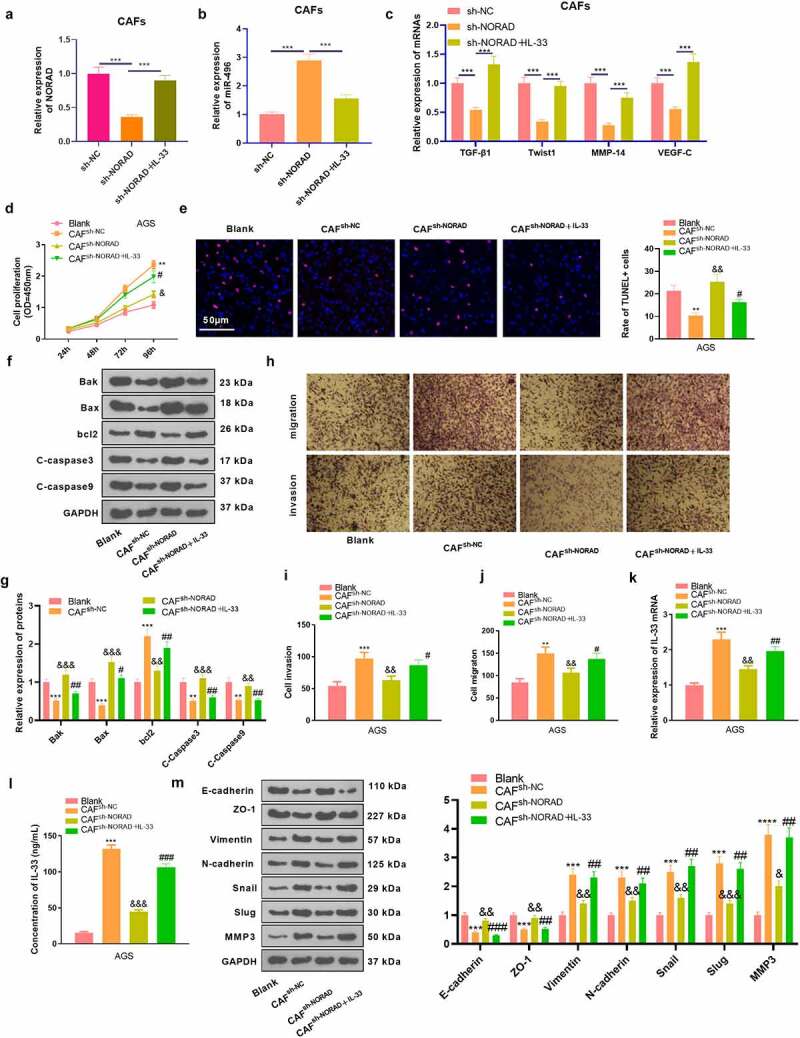
Knocking down NORAD abated CAFs-mediated promotive effects in GC cells. CAFs were transfected with si-NORAD or IL-33 overexpression plasmids. The co-culture model of GC cells with CAFs was constructed to explore the interaction between GC cells and CAF. CAFs (1 × 10^5^/well) were inoculated in the upper chambers, and AGC cells with NORAD knockdown were seeded in 24-well plates (below the chambers) at 5 × 10^5^/well for 24 hours. a-c. RT-PCR evaluated the expression of NORAD, miR-496, and TGF-β1, Twist1, MMP-14 and VEGF-C in CAFs. d.CCK-8 tested the proliferation of AGS cells. E. TUNEL gauged unspecified cell death. f and g. Expression of apoptosis-associated proteins (Bak, Bax, bcl2, Caspase3 and Caspase9) in AGS cells was determined by WB. h-j. Transwell assay was utilized to test the migrative and invasive abilities of AGS cells. k and l. RT-PCR and ELISA were adopted to assess the mRNA and protein level of IL-33 in the supernatant.m. Expression of EMT-related markers (E-cadherin, ZO-1, Vimentin, N-cadherin, Snail, Slug, and MMP3) in AGS cells was evaluated by WB. All data were expressed as mean ± standard deviation. N = 3. **P < 0.01, ***P < 0.001(vs.Blank); &*P* < 0.05, &&*P* < 0.01, &&&*P*< 0.001(vs.CAF^sh-NC^); #*P* < 0.05, ##*P* < 0.01, ###*P*< 0.001(vs.CAF^sh-NORAD^)

To figure out the role of NORAD and IL-33 in CAFs-GC cells interaction, we co-cultured GC cells with CAFs transfected with sh-NC, sh-NORAD, and IL-33 overexpression plasmids. The NROAD-miR-496 level in CAFs were detected. The data showed that NORAD was reduced, whereas miR-496 was increased in CAFs after NORAD knockdown. However, IL-33 overexpression enhanced NORAD, and reduced miR-496 in CAFs Figure 6(a,b). As demonstrated by RT-PCR results, TGF-β1, Twist1, MMP-14 and VEGF-C were markedly up-regulated in CAFs with downregulated NORAD, whereas IL-33 overexpression promoted those mRNAs Figure 6(c). Our data substantiated that proliferation, migration and invasion of GC cells were considerably augmented and apoptosis was restrained in the CAF-sh-NC group (compared to the Blank group). In contrast, knocking down NORAD in CAFs dampened proliferation, migration and invasion of GC cells and contributed to apoptosis, and these effects were attenuated by IL-33 Figure 6(d-j). We then examined the IL-33 expression in GC cells and supernatants, discovering that CAF^sh-NC^ up-regulated IL-33 versus the Blank group, while knockdown of NORAD reduced the IL-33 expression in GC cells. When IL-33 was upregulated in CAFs, GC cells got more IL-33 expression Figure 6(k,l). WB outcomes displayed that by contrast with the Blank group, GC cells in the CAF^sh-NC^ group had diminished expression of E-cadherin and ZO-1 and elevated expression of Vimentin, N-cadherin, Snail, Slug and MMP3. On the contrary, CAF^sh-NORAD^ up-regulated E-cadherin and ZO-1 and down-regulated Vimentin, N-cadherin, Snail, Slug and MMP3 (compared to the CAFsh-NC group), while IL-33 upregulation reversed the effect of NORAD knockdown Figure 6(m). These results supported that knocking down NORAD restrained the interaction between GC cells and CAFs and that IL-33 enhanced the interaction between GC cells and CAFs.

### 3.7 *CAFs stimulated tumor growth* in vivo, *and knocking down NORAD hindered tumor growth*

**Figure 7.**
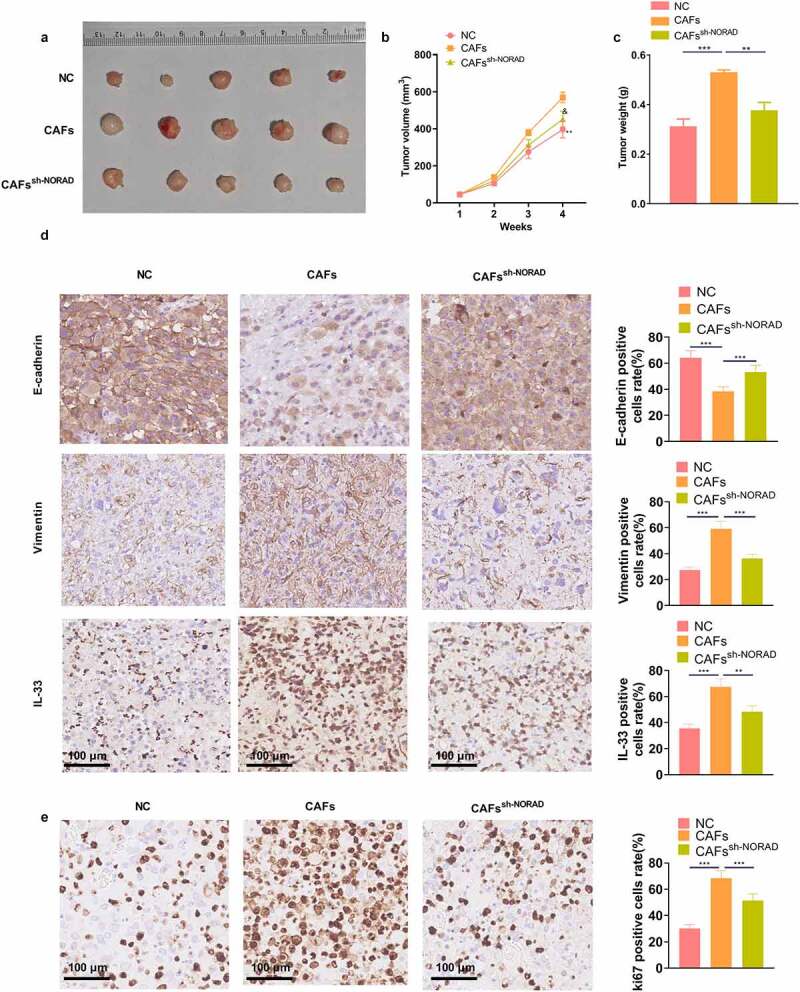
CAFs boosted tumor growth *in vivo*, and knocking down NORAD bridled tumor growth a xenograft model was set up in nude mice, and the volume and mass of the xenograft tumors were measured weekly. b. Representative images of tumors isolated from mice. c. Weight of tumors in nude mice. d and e. The levels of E-cadherin, Vimentin, IL-33 and ki67 in GC tissues were evaluated by IHC. All data were expressed as mean ± standard deviation. N = 5. **P < 0.01, ***P < 0.001 (vs.NC group). &*P* < 0.05(vs.CAFs group)

To characterize the function of NORAD *in vivo*, GC cells co-cultured with CAFs were injected into nude mice to construct a xenograft model. By observing the growth of xenografts, we observed that CAFs enhanced tumor growth *in vivo* versus the NC group, while the mean tumor volume and weight of xenografts in the CAFs^sh-NORAD^ group were correspondingly reduced Figure 7(a-c). The levels of E-cadherin, Vimentin, IL-33 and ki67 in GC tissues were further analyzed by IHC, which corroborated that the positive rates of Vimentin, IL-33 and Ki67 in GC tissues were heightened and the positive rate of E-cadherin was declined in the CAFs group versus the NC group. In parallel, the CAFs^sh-NORAD^ group exhibited a marked reduction in the positivity of Vimentin, IL-33 and ki67 and an increase in the positivity of E-cadherin in GC tissues Figure 7(d,e). These data revealed that CAFs stimulated tumor growth *in vivo*, while knocking down NORAD restrained tumor growth.

## 4 Discussion

**Figure 8.**
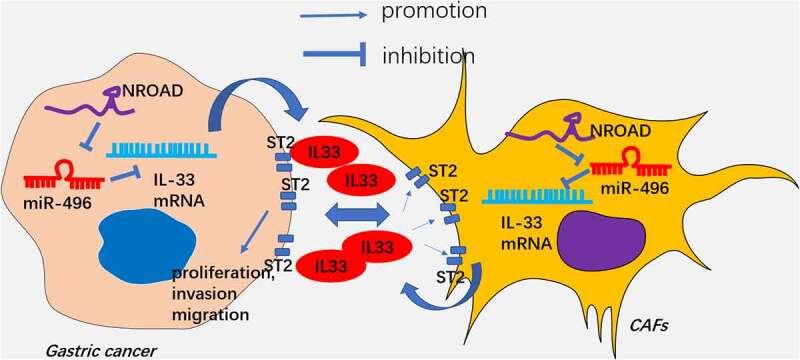
The schematic diagram LncRNA NORAD targets miR-496, and promotes IL-33 in GC cells and CAFs. IL-33 increases GC proliferation, invasion, migration in the GC-CAFs microenvironment

In this study, we explored the role and mechanism of NORAD in GC progression as well as its role in the interaction between GC cells and CAFs. Our results confirmed that NORAD regulated the release of IL-33 by targeting miR-496, thereby expediting GC evolvement. Meanwhile, the NORAD-miR-496-IL-33 axis also controlled the interaction between GC cells and CAFs (Figure 8).

As reported, the dysregulation of lncRNAs plays an essential role in GC tumorigenesis. Those lncRNAs play a role in affecting cell growth, migration, invasion and apoptosis [36–38]. NORAD accelerates the progression of various diseases. For instance, NORAD is down-regulated in the MPP ^+^-mediated Parkinson’s disease model, and overexpressing NORAD weakens the MPP^+^-induced cytotoxicity in SH-SY5Y cells [39]. Moreover, NORAD modulates the development of diabetic nephropathy by mediating toll like receptor 4 [40]. Interestingly, NORAD is dysregulated in different cancer types and is associated with tumorigenesis. NORAD promotes cell proliferation, metastasis and apoptosis through multiple pathways [12–15,41]. Those studies confirmed that NORAD is a vital lncRNA in tumors. Here, we observed up-regulation of NORAD boosted GC cells’ proliferation and invasion and repressed their apoptosis. Those results confirmed the carcinogenic effects of NORAD.

Cytokines and chemokines have a crucial role in mediating CAF-tumor cell interaction in the microenvironment of tumor [42]. Cytokines derived from CAFs dramatically regulate the malignant phenotype of tumor cells. For example, CXCL1 secreted by CAFs modulates radiation-induced DNA damage in a ROS-dependent manner. Overproduced CXCL1 then induces radioresistance in esophageal squamous cell carcinoma [43]. TGFβ and IL-1β can be released by CAFs and can regulate the proliferation, metastasis and invasion of tumor cells [44,45]. CAFs release a large amount of IL-6 in GC and restrain GC cell apoptosis following radiation [46]. Interleukin-33 (IL-33) is a newly discovered member of the IL-1 family, which functions as a transcription factor in the nucleus and plays an immune-regulatory role as an extracellular cytokine [33,34]. A previous study showed that IL-33 is enriched in CAFs and released into the tumor microenvironment. IL-33 promotes M1 to M2 transition of tumor-associated macrophages (TAMs). Mechanistically, IL-33 is recognized by ST2 and activates NF-κB-MMP9-laminin pathway, thus aggravating tumor metastasis [47]. In addition, IL-33 enhances transforming growth factor β (TGF-β) release from macrophages, derives the invasive and drug-resistant properties and further upregulating IL-33 expression of tumor cells [48]. Here, we observed that IL-33 was promoted by NORAD, and knockdown IL-33 reduced GC proliferation and invasion. Besides, CAFs promoted IL-33 expression in GC cells, and IL-33 overexpression reversed sh-NORAD-mediated tumor suppressive effects (Figure 6). Our finding supports that CAFs might regulate the proliferation and invasion of GC cells by up-regulating IL-33.

Multiple miRNAs are implicated in GC development [49,50]. According to reports, miR-496 chokes hypoxia-induced cardiomyocyte apoptosis [51] and restrains non-small cell lung cancer [52]. In this study, we found that miR-496 has potential targeted relationship with NORAD and IL-33. Additional rescue experiments suggested that overexpressing NORAD or IL-33 markedly reversed the anti-tumor role of miR-496 in GC. Thus, NORAD exerts its carcinogenic effect by targeting miR-496 and promotes IL-33 in GC cells (Figure 8).

## 5. Conclusion

This study explored the role and molecular mechanism of mutual regulation between GC cells and CAFs. Our data validated that the lncRNA NORAD-miR-496-IL-33 axis has potential in modulating GC progression and GC-CAFs microenvironment (Figure 8). NORAD, miR-496 and IL-33 are promising diagnostic markers and therapeutic targets in GC. Nevertheless, additional exploration of the role of NORAD-miR-496-IL-33 axis in regulating GC-CAFs interactions *in vivo* is imperative for future studies. and

## Data Availability

The data used to support the findings of this study are available from the corresponding author upon request.
